# Role of *T. cruzi* exposure in the pattern of T cell cytokines among chronically infected HIV and Chagas disease patients

**DOI:** 10.6061/clinics/2017(11)02

**Published:** 2017-11

**Authors:** Tania Regina Tozetto-Mendoza, Dewton de Moraes Vasconcelos, Karim Yaqub Ibrahim, Ana Marli Christovam Sartori, Rita C. Bezerra, Vera Lúcia Teixeira de Freitas, Maria Aparecida Shikanai-Yasuda

**Affiliations:** I Laboratorio de Imunologia (LIM 48), Hospital das Clinicas HCFMUSP, Faculdade de Medicina, Universidade de Sao Paulo, Sao Paulo, SP, BR; II Laboratorio de Virologia (LIM 52), Universidade de Sao Paulo, Instituto de Medicina Tropical, Sao Paulo, SP, BR; III Laboratorio Dermatologia e Imunodeficiencias (LIM-56), Hospital das Clinicas HCFMUSP, Faculdade de Medicina, Universidade de Sao Paulo, Sao Paulo, SP, BR; IV Divisao de Doencas Infecciosas e Parasitarias, Hospital das Clinicas HCFMUSP, Faculdade de Medicina, Universidade de Sao Paulo, Sao Paulo, SP, BR; V Departamento de Doencas Infecciosas e Parasitarias, Faculdade de Medicina FMUSP, Universidade de Sao Paulo, Sao Paulo, SP, BR; VI Laboratorio de Parasitologia (LIM 46), Hospital das Clínicas HCFMUSP, Faculdade de Medicina, Universidade de Sao Paulo, Sao Paulo, SP, BR

**Keywords:** Intracellular Cytokines, Chagas Disease, HIV, Trypomastigote Antigen, T Cells

## Abstract

**OBJECTIVES:**

The impact of Chagas disease (CD) in HIV-infected patients is relevant throughout the world. In fact, the characterization of the adaptive immune response in the context of co-infection is important for predicting the need for interventions in areas in which HIV and Chagas disease co-exist.

**METHODS:**

We described and compared the frequency of cytokine-producing T cells stimulated with soluble antigen of *Trypanosoma cruzi* (*T. cruzi*) using a cytometric assay for the following groups: individuals with chronic Chagas disease (CHR, n=10), those with Chagas disease and HIV infection (CO, n=11), those with only HIV (HIV, n=14) and healthy individuals (C, n=15).

**RESULTS:**

We found 1) a constitutively lower frequency of IL-2+ and IFN-γ+ T cells in the CHR group compared with the HIV, CO and healthy groups; 2) a suppressive activity of soluble *T. cruzi* antigen, which down-regulated IL-2+CD4+ and IFN-γ+CD4+ phenotypes, notably in the healthy group; 3) a down-regulation of inflammatory cytokines on CD8+ T cells in the indeterminate form of Chagas disease; and 4) a significant increase in IL-10+CD8+ cells distinguishing the indeterminate form from the cardiac/digestive form of Chagas disease, even in the presence of HIV infection.

**CONCLUSIONS:**

Taken together, our data suggest the presence of an immunoregulatory response in chronic Chagas disease, which seems to be driven by *T. cruzi* antigens. Our findings provide new insights into immunotherapeutic strategies for people living with HIV/AIDS and Chagas disease.

## INTRODUCTION

Chagas disease (CD), caused by the protozoan *Trypanosoma cruzi (T. cruzi)*, which is transmitted to humans by a vector (reduviid bugs), is currently one of the most important neglected diseases in Latin America. After the control of transmission by *Triatoma infestans,* the main vector in Brazil, the most important route is oral transmission associated with acute cases and outbreaks 1. Migration from rural areas has made chronic CD primarily an urban disease in Latin America and the United States 1-6. Transmission of CD through blood transfusion, blood by-products, or organ transplantation is currently a serious problem in non-endemic areas. At least 5 to 6 million chronically infected individuals live in endemic and non-endemic countries, and the disease continues to represent a health threat around the world 6.

Acute CD is characterized by alterations in the mononuclear phagocytic system, causing lymphadenopathies and, less frequently, severe myocarditis or meningoencephalitis. Additionally, although most chronically infected individuals are asymptomatic, approximately 30 to 40% develop recognized cardiomyopathy or gastrointestinal tract disorders 7.

Reactivation of CD manifests as a febrile syndrome with meningoencephalitis and/or myocarditis, which is also associated with HIV infection and other immunodeficiency states such as haematological malignancies, bone marrow, kidney, or heart transplantation, and corticosteroid therapy 8-11. Reactivation of CD in AIDS patients has been observed in ≤20% of co-infected patients and has sometimes been reported as the first opportunistic infection 12. According to Almeida et al. 2011 13, the overall mortality rate of HIV patients was 30%, and mortality occurred in 73% of the cases in which there was reactivation of CD.

The frequency of individuals co-infected with HIV and *T. cruz*i was estimated at 1.3% in a Brazilian study with a macro-regional approach 14. The impact of co-infection is extremely relevant, as approximately 1.5 million individuals with HIV/AIDS live in Latin America and approximately 21,420 HIV/*T. cruzi* co-infected patients are estimated to live in this area 15.

Immunoregulatory mechanisms may influence the pathogenesis and clinical evolution of CD 16. Because CD and HIV infection are both associated with T cell responses and disturbances of cytokine networks 17, 18, the characterization of cytokine-secreting T cells is particularly relevant to improving our understanding of the immunopathogenesis of CD and to controlling concomitant intracellular infections in AIDS and other immunosuppressive conditions. A study of the differential regulation of Th1 and Th2 responses in HIV infection showed that decreased secretion of type-1 cytokines, such as IL-2 and IFN-γ, was associated with a higher susceptibility to opportunistic infections 19. Conversely, previous studies of the pathogenesis and clinical evolution of CD have reported higher IL-4/IFN-γ ratios in patients with HIV/Chagas disease as well as the preferential involvement of inflammatory cytokines and activated T cells 18, 20. However, it is unclear whether the presence of HIV and *T. cruzi* co-infection modifies this mechanism in humans.

Recent studies have demonstrated the impact of a specific antigenic stimulus on the course of a chronic infection in mice, as seen in the association between an HIV vaccine and helminthic infection 21. Accordingly, the characterization of the adaptive immune response either in mouse models or in human infections is relevant to interpreting or predicting therapeutic interventions in endemic areas where HIV and other infections co-exist.

This study aimed to describe and compare the profiles of cytokine-producing T cells from individuals with chronic Chagas disease and/or HIV infection with those from healthy individuals using a cytometric assay, which detects the intracellular accumulation of cytokines in CD4+ and CD8+ T lymphocytes stimulated with soluble trypomastigote antigens and mitogens.

## MATERIALS AND METHODS

### Ethics Statement

The Human Research Ethics Committee of the Hospital das Clínicas, Faculdade de Medicina, University of São Paulo approved the research protocol (CAPPesp 010/95-B). A signed informed consent form was obtained from all 50 participants (35 patients diagnosed with CD and/or HIV infection and 15 healthy individuals) for the period of 2001-2005 to participate in the present cross-sectional study based on convenience sampling.

### Study Groups and Methods

We enrolled 35 patients diagnosed with chronic CD and/or HIV infection who attended the Outpatient Clinic, Division of Clinics of Infectious and Parasitic Diseases of the Hospital das Clínicas, Faculdade de Medicina, University of São Paulo. HIV infection was diagnosed with ELISA and confirmed with Western blotting. CD4+ and CD8+ T cell counts were measured by flow cytometry, and HIV viral load was determined by polymerase chain reaction (PCR). All HIV-infected individuals received Highly Active Antiretroviral Therapy (HAART), according to the national guidelines for antiretroviral therapy 15. For CD diagnosis (the CHR group), seropositivity in at least two of three tests for *T. cruzi* infection (i.e., ELISA, indirect immunofluorescence assay, and indirect haemagglutination assay) was required. The clinical forms of CD were classified according to Sartori et al. 2007 22: (1) “**Indeterminate form**” (IND), meaning no symptoms or signs and normal results for electrocardiogram (ECG), chest X-ray, and oesophagography. (2) “**Non-typical cardiopathy**” or “**atypical manifestations**” (AT), meaning ECG changes possibly related to conditions other than CD. (3) “**Typical cardiopathic form**” with ECG changes considered typical of CD, including sinus bradycardia <50 beats/min, right bundle-branch block, second-degree atrioventricular block, and/or complex ventricular arrhythmia. (4) “**Digestive form**”, meaning megaoesophagus or megacolon. (5) “**Typical digestive and cardiopathy**” (TDC), meaning typical cardiac and/or digestive manifestation. Xenodiagnosis 23 and blood culture 24 were requested according to medical orientation and performed by the Laboratory of Parasitology of the Clinics Hospital. Blood culture and/or xenodiagnosis data were used to confirm the presence or absence of parasites (*T. cruzi*) or reactivation of infection by *T. cruzi* at the time of this study. Qualitative PCR was performed using primer pairs for S35 and S36 kinetoplast sequences 25. Qualitative PCR was also performed using TCZ3/TCZ4 microsatellite sequences 26.

Therefore, we analysed four groups. There were 10 individuals with chronic CD and not infected with HIV (CHR group), 11 with chronic CD and HIV infection (CO group), 14 with HIV infection without CD (HIV group) and 15 healthy individuals (C group). The C group consisted of individuals reporting the absence of CD, HIV diagnosis or any other chronic or acute illnesses, with CD4+ and CD8+ levels above those associated with susceptibility to opportunistic diseases (>500 cells/mm^3^) and aged <50 years old.

We assessed the frequencies of CD4+ or CD8+ T cells staining positive for IL-2, IFN-γ, IL-4, and IL-10 after being incubated *in vitro* with SAg and/or mitogens (phorbol and ionomycin), according to the methods described below.

Concerning the available data on the clinical form of CD in individuals with or without HIV infection, we compared two sub-groups of CD: typical digestive and/or cardiac manifestation (TDC) (n=8) and indeterminate form (IND) (n=8). Two patients with an atypical clinical (AT) form of CD and three with an unknown or non-identified (NI) form of CD were excluded from these analyses.

### Cell Preparation and *In Vitro* Stimulation with Soluble Antigen of *T. cruzi* Trypomastigote (SAg)

Samples of peripheral blood mononuclear cells (PBMCs) were isolated from heparinized venous blood (30 mL) using density gradient sedimentation (1.077 g/mL) in accordance with the recommendations of the manufacturer (Ficoll-Paque - Amersham Bioscience, Piscataway, NJ, USA). The cells were then washed three times with sterile phosphate-buffered saline (PBS) without Mg++/Ca++ (Gibco BRL, UK) and re-suspended at 2.5 x 10^6^ cells/mL in RPMI 1640 (Gibco BRL, UK) supplemented with 10% heat inactivated foetal calf serum (HyClone, Logan, Utah, USA), 2 mM L-glutamine (Gibco BRL, UK) and antibiotics, according to Sousa e Victorino, (1998) 27 for *in vitro* stimulation and immunofluorescence staining of freshly isolated cells.

The soluble antigen obtained by complete disruption of 1.0 x 10^6^ parasites/mL (10 cycles of freezing and thawing at the trypomastigote stage) was kindly provided by Dr. Ises de Almeida Abrahamsohn (Department of Immunology of the Institute of Biomedical Sciences of the University of São Paulo, SP, Brazil).

Two different antigenic stimuli were used to stimulate cells *in vitro,* according to De Barros-Mazon et al. (2004) 20, but with some modifications. One set of cells was stimulated with specific soluble trypomastigote antigen (SAg) for 72 hours followed by brefeldin A (BFA, Sigma Chemical) at a final concentration of 10 µg/ml and phorbol 12-myristate 13-acetate (PMA, Sigma Chemical) + ionomycin (I, Sigma Chemical) at a final concentration of 50 ng/mL and 500 ng/mL, respectively, for the last 4 hours. In addition, another sample of cells was subjected to the same protocol with only PMA+I+BFA and not SAg to measure the potential capability of each cell to produce these cytokines. It has been shown that following stimulation of lymphocytes with PMA and ionomycin, a rapid down-regulation of CD4 molecules on the surface of lymphocytes occurs 28. In our hands, a rapid decrease in CD4 occurred within 4 hours after stimulation. Therefore, a negative-gating strategy (among CD3+ T cells, CD8- cells were selected) was used to measure the intracellular cytokine expression in CD3+CD4+ cells. To normalize the comparison of inter-assay results, each assay analysed PBMCs from individuals in the four groups, with at least one individual of each group (C, CO, HIV and CHR) measured simultaneously.

### Monoclonal Antibodies (MoAbs) for Intracellular and Cell Surface Staining

The following phycoerythrin-conjugated anti-human cytokines and corresponding isotype controls were used for each antibody and each patient: anti-IL-2, clone M062626; anti-IFN-γ, clone M064882; anti-IL-4, clone M064843; anti-IL-10, clone M063277; rat IgG2a, clone M063752; and mouse IgG1, clone M056389. Surface staining was performed with anti-CD3 phycoerythrin-cyanin 5 (PE-Cy5), clone M047602, and anti-CD8 fluorescein isothiocyanate (FITC), clone M055034. All antibodies were purchased from Pharmingen (San Diego, CA).

### Immunofluorescence Staining and Flow Cytometric Analyses

Cells were washed in PBS with 0.1% sodium azide (PBS-azide). First, the Fc receptors of the cells were blocked with 2 µg of human IVIg (50 µg/ml) and incubated for 20 minutes at room temperature to decrease non-specific staining. Next, the cells were washed in PBS-azide and re-suspended in PBS (Gibco, without Ca+ and Mg+) with 1% bovine serum albumin (BSA, Sigma) and 0.1% sodium azide (Azide) (staining buffer). After surface staining with anti-CD8 and anti-CD3 MoAbs, the cells were washed with staining buffer and fixed with PBS-1% paraformaldehyde (fixation buffer). These steps were performed at 4°C in the dark. Subsequently, the cells were permeabilized with PBS with 1% BSA and 0.5% saponin (permeabilization buffer). Intracellular staining was performed by incubation with anti-IL-2, anti-IL-4, anti-IL-10, and anti-IFN-γ MoAbs or respective isotype controls for 30 minutes at room temperature in the dark. Next, the cells were washed and re-suspended in PBS-azide for flow cytometric analysis.

**Figure 1:**
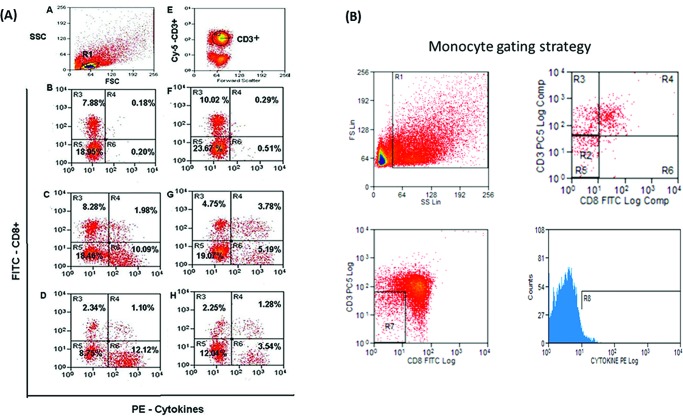
Flow cytometric evaluation. (A) *T. cruzi*-stimulated cells. Lymphocytes in the R1 region based on the SSC/FSC (Side Scatter/Forward Scatter) ratio; (E) CD3 surface marker staining on lymphocytes gated in R1; (B, C, D, F, G, H) CD8 surface- and intracellular IFN-γ-stained cells were simultaneously gated in R1 (lymphocytes) and R2 (CD3+ cells). R3 and R5 regions represent CD8+ and CD8- (i.e.: CD4+) cells negative for IFN-γ, respectively; R4 and R6 represent CD8+IFN-γ+ and CD4+IFN-γ+ cells, respectively. Peripheral blood mononuclear cells were cultured for 72 h without *T. cruzi* antigen (B, C, D) and with *T. cruzi* antigen (F, G, H) and subsequently exposed to PMA, ionomycin, and brefeldin A (BFA) for the last 4 h. For each analysis, approximately 50,000 events were acquired on a FACSCalibur flow cytometer. (B) Monocyte gate strategy. Flow cytometric acquisition and subsequent analysis were manually performed within a monocyte gate defined for each sample using CellQuest (BD Bioscience, Immunocytometry System) based on a higher FSC/SSC ratio and negative expression of CD3 and CD8 (R7 region) and are displayed as histograms showing cytokines (PE) (R8 region).

For each analysis, approximately 50,000 events were acquired on a FACSCalibur flow cytometer (Becton-Dickinson, San Jose, CA, USA). Flow cytometric acquisition was performed within a lymphocyte gate defined for each sample using CellQuest (BD Bioscience, Immunocytometry System) and displayed as dot plots showing cytokines (PE) *versus* CD3 (PE-Cy5) and CD8 (FITC) fluorescence in multiple dot plots. The CD8 dot plot was analysed for double dependence and CD3 expression on an FSC-SSC lymphocyte gate (Figure 1, Figure 1A). The monocyte gate was determined by FSC-SSC and exclusion of CD3+ and CD8+ populations (Figure 1, Figure 1 B).

### Statistical Analysis

For comparisons between two groups, the Mann-Whitney test was used. For within-group comparisons, the Wilcoxon test was used. For comparisons among three groups (HIV, C and CHR), the Kruskal Wallis test was used. Statistical significance was set at 5% (*p*<0.05). The Spearman correlation coefficient was used to measure bivariate associations of the proportion of cytokines.

## RESULTS

### Clinical and Laboratory Data of the Study Groups

Data regarding the clinical groups at the time of enrolling in the study are shown in Table 1.

The median age distribution was 30, 40, 47 and 42 years old for the C, HIV, CO and CHR groups, respectively. The proportion of male participants was lower in the CHR group (30.0%) than in the CO group (54.5%) and HIV group (78.6%), but the differences did not reach statistical significance among these groups (Kruskal Wallis test, *p*=0.9639). There was an equal proportion of females in the CHR group and C group (70-73%). Moreover, there was no difference in the CD4+ cell count among the HIV, CO and CHR groups (Kruskal Wallis test, *p*=0.0921) (Table 1A).

The distribution of CD sub-groups and the mean CD4+ cell count among individuals with positive (n=4) and negative parasitaemia (n=11) detected by haemoculture, xenodiagnosis and PCR were also presented. PCR analysis for parasite DNA fragments was positive in two chagasic individuals with HIV infection (CO group) of eight available plasma samples. The CD4+ cell count tended to be higher in individuals with negative parasitaemia than with positive parasitaemia, but without statistical significance (Table 1B).

### Comparison of the Phenotype Frequency of Cytokines on CD4+ and CD8+ T cells from the C, HIV, CO, and CHR Groups

#### In the Absence of SAg Antigen

**Figure 2:**
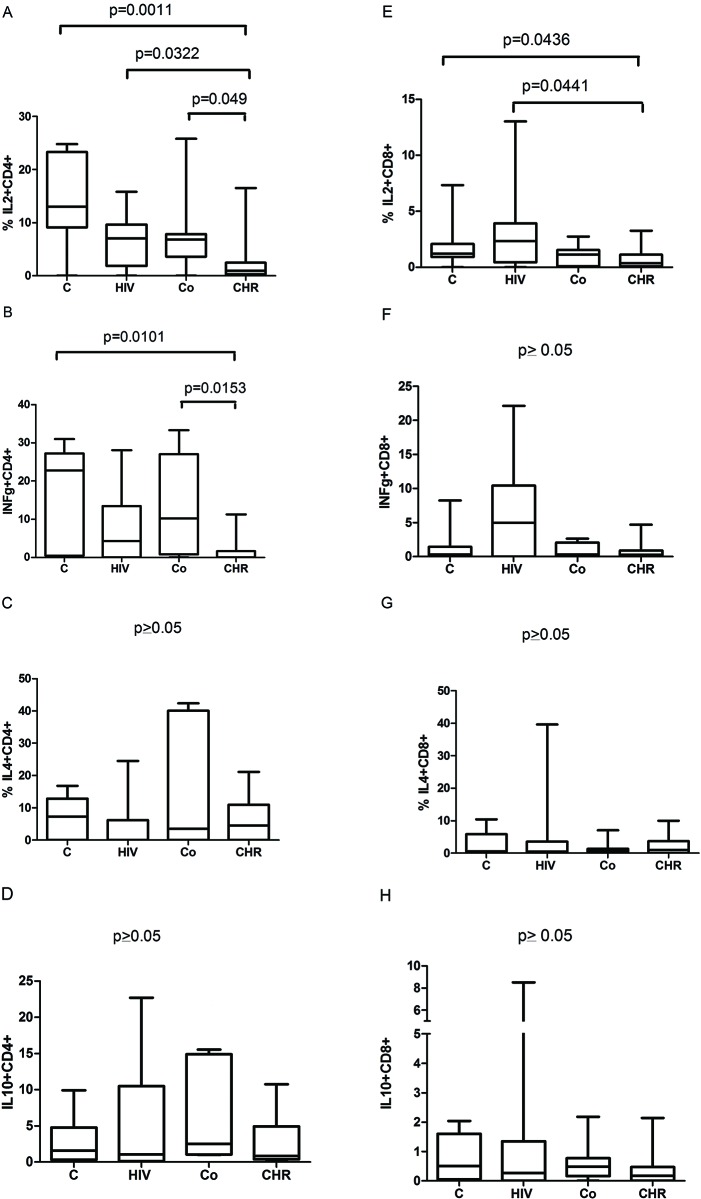
Comparison of the potential capability of T cells to produce cytokines. (A-D) Proportion of cytokines on CD4+ T cells. (E-H) Proportion of cytokines on T CD8+ T cells. C = healthy controls; HIV = HIV infection; CO = co-infected with Chagas disease and HIV; CHR = chronic Chagas disease. IL-2+CD4+ phenotype: a statistically significant difference was observed between the CHR and HIV groups (Mann-Whitney test, *p*=0.0322), the CHR and C groups (Mann-Whitney test, *p*=0.0011), and the CHR and CO groups (Mann-Whitney test, *p*=0.0049). IL-2+CD8+ phenotype: a statistically significant difference was observed between the CHR and HIV groups (Mann-Whitney test, *p*=0.0441) and between the CHR and C groups (Mann-Whitney test, *p*=0.0436). IFN-γ+CD4+ phenotype: a statistically significant difference was observed between the CHR and C groups (Mann-Whitney test, *p*=0.0101) and between the CHR and CO groups (Mann-Whitney test, *p*=0.0153). No statistically significant differences were observed in relation to the other phenotypes.

The potential capacity of production of IL-2+ and IFN-γ+ T cells in the CHR group was low, as determined by comparing the CHR group with each other group (C, HIV, CO) and shown in Figure 2 (Figure 2A, 2B, 2E). No significant differences were found in other cellular phenotypes (Figure 2C, 2D, 2F, 2G, 2H).

#### In the Presence of In Vitro SAg Stimulation

**Figure 3:**
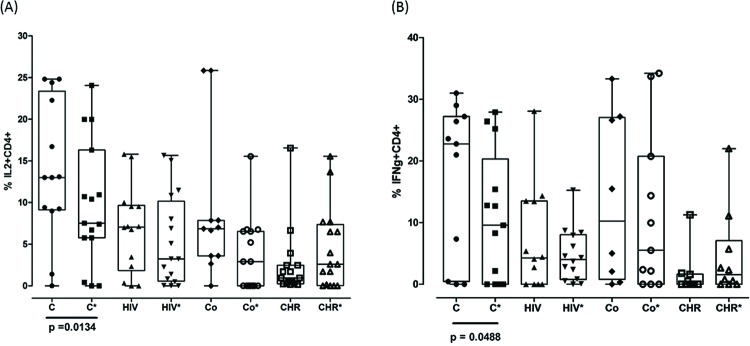
Comparison of the proportion of cytokines on T cells with and without SAg stimulus. (A). IL-2+CD4+. (B) IFN-γ+CD4+. The asterisk (*) represents the condition in which cells were stimulated with soluble *T. cruzi* antigen. The difference between non-stimulated and stimulated* cells was significant for IL-2+CD4+ (Wilcoxon test, *p*=0.0093) and IFN-γ+CD4+ T cells (Wilcoxon test, *p*=0.048). No statistically significant differences were observed in relation to the other phenotypes.

When we compared cells stimulated and non-stimulated with SAg from healthy individuals (group C) in particular, we observed a reduced proportion of IL-2+CD4+ and IFN-γ+CD4+ cells in the presence of SAg, as shown in Figure 3 (Figure 3A, Figure 3B). We found no significant differences when we compared other T cell phenotypes from the CO, CHR and HIV groups in the presence or absence of SAg.

### Comparison between the Indeterminate Form and Typical Clinical Manifestation of CD

**Figure 4:**
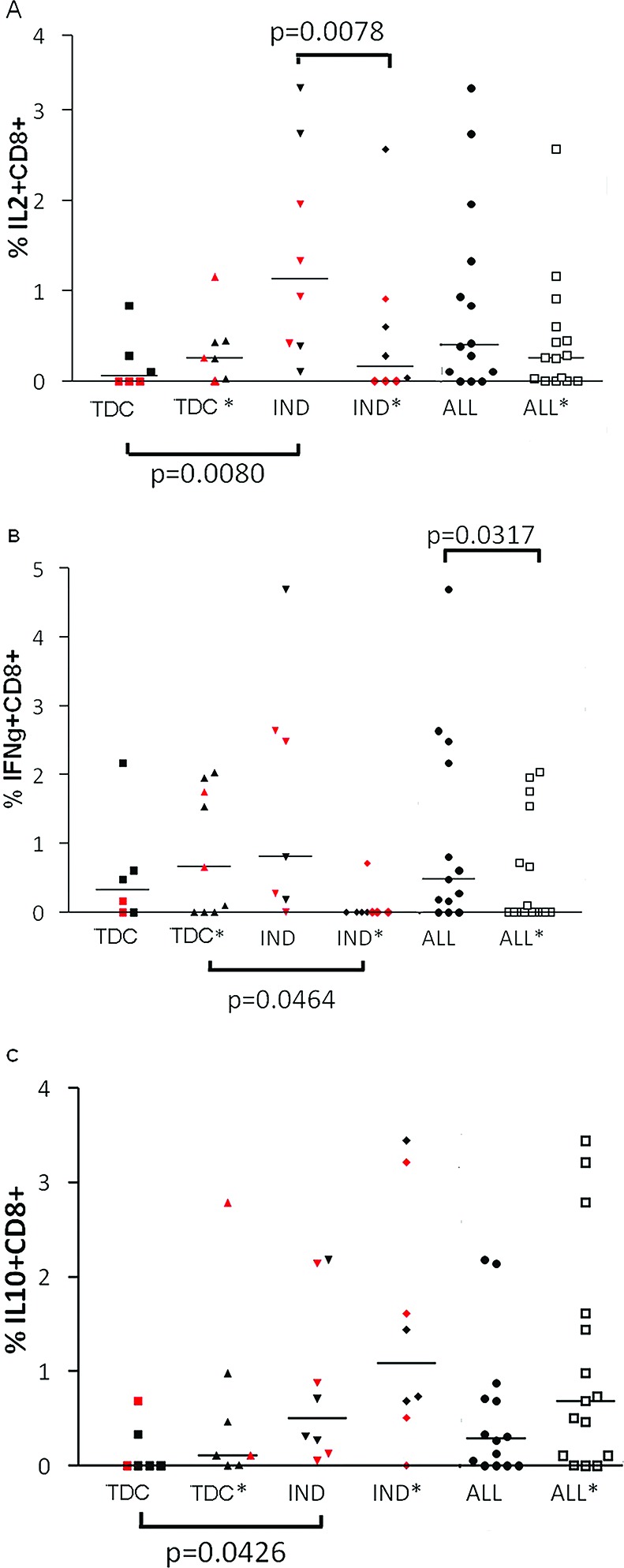
Proportion of cytokine-producing CD8+ T cells in chagasic sub-groups. (A) IL-2+CD8+. (B) IFN-γ+CD8+. (C) IL-10+CD8+. Patients were subdivided into a typical digestive and/or cardiac manifestation (TDC) sub-group and a non-cardiac/non-digestive manifestation of CD (IND) sub-group, and all chagasic (TDC and IND) patients were identified as the ALL group. The asterisk (*) represents the condition in which cells were stimulated with soluble *T. cruzi* antigen. The TDC and IND sub-groups had four HIV-infected individuals each. Red plots indicate HIV-infected individuals. The Mann-Whitney test was used for between-group comparisons (p indicated below the x-axis), and the Wilcoxon test was used for within-group comparisons (p indicated above the x-axis). The difference between TDC* and IND* stimulated T cells to produce cytokines was significant for the IFN-γ+CD8+ phenotype (Mann-Whitney test *p*=0.0464). The potential capability of T cells to produce cytokines was different between TDC and IND for the IL-2+CD8+ phenotype (Mann-Whitney test, *p*=0.0080) and the IL-10+CD8+ phenotype (Mann-Whitney test, *p*=0.0426). No significant differences were found in relation to the other phenotypes.

The results indicated a differential frequency of cell phenotypes between the IND and TDC sub-groups of CD (n=16), as shown in Figure 4.

In Both the TDC (n=8) and IND (n=8) sub-groups, half of the patients were HIV-infected patients with low or undetectable viral load. In addition, six positive parasitological tests were detected in the sub-groups of CD.

In the absence of SAg, the frequencies of IL-2+CD8+ and IL-10+CD8+ phenotypes were higher in sub-group IND than in sub-group TDC (Mann-Whitney test, *p*=0.0080 and 0.0426, respectively). Conversely, the frequency of the IL-2+CD8+ phenotype in IND was lower in the absence of SAg than in the presence of SAg (IND and IND*, Wilcoxon test, *p*=0.0078). Furthermore, in the presence of cellular stimulation by SAg, the proportion of IFN-γ+CD8+ cells was lower in IND than in TDC (Mann-Whitney test, *p*=0.0464). Similarly, we observed that the frequency of this phenotype was significantly lower in all chagasic individuals, even in those co-infected with HIV (ALL and ALL*, Mann-Whitney test, *p*=0.0317, Figure 4).

We also found a negative correlation between IFN-γ and IL-10 (*p*=0.0390; r=-0.6691) when the source of cytokines was CD8+ T cells derived from chronic chagasic patients.

## DISCUSSION

We have described the profile of intracellular cytokine production in T cells from HIV-infected and/or chagasic individuals by focusing on three important aspects, showing that *T. cruzi* exposure played a pivotal role in the down-regulation of inflammatory cytokine-producing T cells in human infections.

First, we showed that the potential capability of T cells to produce IL-2 and IFN-γ was lower in individuals with chronic CD compared with each of the other groups analysed (HIV, CO or C). IL-2 has been described as a prototypic Th1 cytokine, being essential for the differentiation and activation of regulatory T cells, and as noted here, the decreased IL-2 production in chronic CD seems to suggest that an adaptive activation mechanism is triggered by prior exposure to *T. cruzi* infection (Figure 2, Figure 2A, 2B and 2E). The decreased inflammatory profile could be associated with the pathogenesis of cardiac and gastrointestinal diseases, since the immunoregulatory features seem to be linked to the indeterminate form of CD 29, 30.

Second, we observed *in vitro* an immunomodulatory effect of SAg, mainly expressed in cells from healthy individuals. In fact, a significant down-regulation of IL-2+CD4+ and IFN-γ+CD4+ cells was observed in healthy individuals (group C), who reported the absence of acute or chronic disease and did not belong to the risk group exposed to *T. cruzi* or HIV infection (Figure 3, Figure 3A, Figure 3B). We observed the absence of this effect in the CHR group, as these cell phenotypes were already low at background level in these patients. Corroborating our data, other protocols based either on soluble antigen or on cell cultures stimulated by living trypomastigotes suggest that this effect of the HIV-*T. cruzi* interaction on cytokine secretion is parasite driven 31, 32. By contrast, protocols based on cellular stimulation with epimastigotes or recombinant B13 antigen do not seem to induce a suppressive immune response like the response observed in this study using trypomastigote antigen 20.

Third, despite the limited sample size, we observed a polarized Th1 CD8+ T cell response when cells from chagasic individuals were exposed *in vitro* to SAg stimuli, thus distinguishing the typical digestive/cardiac form (TDC group) from the indeterminate form of CD (IND group) (Figure 4). In the TDC group, we clearly verified an up-regulation of inflammatory cytokines on CD8+ T cells challenged with soluble trypomastigote antigen. However, the biased inflammatory response characterizing individuals with the cardiac and/or digestive form of CD 33 could be justified by the absence of the activation of a regulatory immune mechanism. Moreover, the profile of cytokines observed in the IND sub-group has been previously suggested to be a strategy that improves the chances of parasite survival 28. Additionally, CD8+ T cells seem to play a role in the balance between pro-inflammatory responses to eradicate the pathogen and anti-inflammatory responses to limit inflammation as described in CD and other infections 34,35. In fact, the TDC sub-group presented a lower frequency of IL-10+CD8+ cells and a higher frequency of IFN-γ+CD8+ cells than individuals with the indeterminate form of CD (IND). This was especially true for individuals with HIV infection (Figure 4, red points).

Nevertheless, studies on the source of IL-10 in humans are conflicting, partly due to the different assessment methods used 36,37,38. Thus, we also assessed the production of IL-10 by monocytes with and without SAg stimulation, but we did not find any significant difference between the clinical groups (data not shown). On the other hand, we were able to detect a significant difference between the IND and TDC sub-groups in relation to the IL-10+CD8+ phenotype. Our findings corroborate the fact that IL-10 produced by T cells seems to act mainly as a regulatory cytokine to protect the infected hosts against unwanted excessive Th1 activation and severe inflammatory pathology 20,29,34.

Our patients exhibited a trend of higher IL-4 production by SAg-stimulated CD4+ T cells in the co-infected (CO) group compared with the HIV group, similar to the patients studied by Rodrigues et al. (2005) 18. The percentage of IL-4-producing CD4+ T cells stimulated by SAg varied from zero to 27.47% with a median of 10.71% in the CO group, while in HIV group, the same cell population was between zero and 46.79% with a median of 0.0% (*p*=0.0957).

Moreover, the same tendency was noted by comparing the CO and CHR groups, as the former tended to show higher percentages of SAg-stimulated IL-4-producing CD4+ T cells than the latter. The percentage of IL-4-producing SAg-stimulated CD4+ T cells varied from zero to 27.47% with a median of 10.71% in the CO group, while that of the CHR group varied from zero to 20.84% with a median of 4.24% (*p*=0.1108). This trend was not observed when we compared the non-SAg-stimulated CD4+ T cells of the CO group with those of the HIV or CHR groups. These data suggest that SAg can induce the synthesis of IL-4 and that the co-infected group is more sensitive to this stimulus.

Recently, studies have characterized the adaptive immune response in different epidemiologic contexts. In fact, the interference of previous exposure to a specific antigen (vaccinated or not) has been reported in both dual infections or other models of infections involving HIV. In the infection model of *Schistosoma* in mice, the suppression of the immune response to a Th1-type HIV vaccine and impaired expansion of pathogen-specific cytotoxic CD8+ T cell responses was reported 21. In addition, in children living with HIV, higher CD4+ T cell activation leads to poor vaccine response, which may be related to a disequilibrium of T regulatory responses 39, 40. In infant macaques vaccinated with BCG or *M. tuberculosis*, CD4+ T cells were persistently activated in oral and/or gastrointestinal tissues, which may have facilitated oral SIV infection, according to the authors 41. Moreover, in HIV-infected Kenyan infants, higher central memory Th1 responses to *M. tuberculosis* antigens were observed at three months, but reduced effector memory Th1 responses to vaccine antigens were seen at three and 12 months. Long-term monitoring of vaccine efficacy and T-cell immunity in this vulnerable population is warranted 42.

Our study, however, has some important limitations. First, because it was not possible to obtain serial CD4+ cell counts, HIV viral loads or parasitological data, it would be very useful to verify additional relationships with immune responses. This fact is directly linked to the limited access patients with CD have to medical assistance and diagnosis. Second, our critical findings were based on a limited number of available samples. However, we screened a rare and well-characterized group of HIV and/or chagasic individuals.

## AUTHOR CONTRIBUTIONS

Tozetto-Mendoza TR and Vasconcelos DM were responsible for writing and revising the manuscript, performing the cellular assays, analysing the data and supervising the study. Ibrahim KY and Sartori AM were responsible for the clinical analysis of the participants and paper revision. Bezerra RC and de Freitas VL were responsible for performing the parasitological tests. Shikanai-Yasuda MA was responsible for the study coordination and for writing and critically revising the manuscript.

## Figures and Tables

**Table 1A:** Study groups according to CD4+ cell count, age, sex and sub-groups of Chagas disease.

Variables	groups	C	HIV	CO	CHR
	n	15	14	11	10
CD4+ count /cells/mm^3^	median (min-max)	961 (933-988)	475 (243-870)	505 (218-837)	855 (551-1198)
Age	years (median)	30	40	47	42
Female	%	73.0	21.4	45.5	70.0
Subgroups of CD					
TDC	n	NA	NA	4	4
IND	n	NA	NA	4	4
AT	n	NA	NA	2	0
NI	n	NA	NA	1	2

min=minimum; max=maximum; n=number of individuals; CD=Chagas disease; C=healthy controls; HIV=HIV infection; CO=co-infected with Chagas disease and HIV; CHR=chronic Chagas disease; TDC=typical digestive and/or cardiac involvement; IND=indeterminate form of CD; AT=atypical form of CD; NI=not identified; NA=not applied; (SD)=standard deviation; n=number of patients.

**Table 1B:** CD4 cells/mm^3^ in 15 Chagas disease individuals (ALL) according to the presence of parasitaemia (detected by PCR, haemoculture and xenodiagnoses).

ALL Chagas disease	Positive parasitemia	Negative parasitemia
**Disease form (Number of Patients)**	n	n
CO and IND	1	3
CO and TDC	1	4
CHR and IND	1	2
CHR and TDC	1	1
CHR and NI	0	1
**HIV viral load (copies/mL)**		
Mean (SD)	3.6 (3.5)^a^	4.3 (4.1)^b^
Indetectable (%)	25.0	66.7
**Mean (SD) CD4 cells/mm^3^**	361 (166)^c^	680 (278)^c^
Total Number of Patients	4	11

CD=Chagas disease; C=healthy controls; HIV=HIV infection; CO=co-infected with Chagas disease and HIV; CHR=chronic Chagas disease; TDC=typical digestive and/or cardiac involvement; IND=indeterminate form of CD; AT=atypical form of CD; NI=not identified; (SD)=standard deviation; n=number of patients.

a two with available HIV viral load data.

b six with available HIV viral load data.

c *p*>0.05 between CD4+ cells of positive and negative parasitaemia patients (Mann-Whitney test).
